# A Cross-Sectional Assessment of Parental Concerns in the Pediatric Surgery Department during the COVID-19 Pandemic

**DOI:** 10.3390/healthcare11091330

**Published:** 2023-05-05

**Authors:** Ada Claudia Silvana Gruescu, Calin Popoiu, Mihaela Codrina Levai, Raluca Tudor, Roxana Manuela Fericean, Mircea Rivis

**Affiliations:** 1 Department of Pediatrics, Victor Babes University of Medicine and Pharmacy Timisoara, E. Murgu Square, Nr. 2, 300041 Timisoara, Romania; ada_gruescu@yahoo.com (A.C.S.G.); mcpopoiu@umft.ro (C.P.); 2 Doctoral School, Victor Babes University of Medicine and Pharmacy Timisoara, E. Murgu Square, Nr. 2, 300041 Timisoara, Romania; manuela.fericean@umft.ro; 3 Research Center for Medical Communication, Victor Babes University of Medicine and Pharmacy Timisoara, E. Murgu Square, Nr. 2, 300041 Timisoara, Romania; codrinalevai@umft.ro; 4 Second Discipline of Neurology, “Victor Babes” University of Medicine and Pharmacy Timisoara, Eftimie Murgu Square 2, 300041 Timisoara, Romania; 5 Department of Infectious Diseases, “Victor Babes” University of Medicine and Pharmacy, Eftimie Murgu Square 2, 300041 Timisoara, Romania; 6 Department of Anesthesiology and Oral Surgery, Multidisciplinary Center for Research, Evaluation, Diagnosis and Therapies in Oral Medicine, “Victor Babes” University of Medicine and Pharmacy Timisoara, Eftimie Murgu Square 2, 300041 Timisoara, Romania; rivis.mircea@umft.ro

**Keywords:** COVID-19, pediatric surgery, SARS-CoV-2, parental stress

## Abstract

The COVID-19 pandemic has impacted various aspects of healthcare, including pediatric surgery. This study aimed to assess parental concerns and stress levels in pediatric surgery during the COVID-19 pandemic, identify factors associated with increased parental anxiety or concern, and provide recommendations for healthcare providers. A cross-sectional study was conducted in a tertiary pediatric hospital in Timisoara, Romania, involving 174 parents of pediatric patients requiring elective or emergency surgery, with a mean age of 37.6 (25–47) years, out of which 89.1% of respondents were women. Parental concerns were assessed using the Parental Concerns Questionnaire (PCQ), the Hospital Anxiety and Depression Scale (HADS), and the Perceived Stress Scale (PSS-10). Parents of children undergoing emergency surgery (*n* = 108) reported higher levels on the practical impact domain of the PCQ scale (3.4 vs. 2.2, *p* < 0.001), emotional impact (2.7 vs. 2.2, *p* = 0.002), and total PCQ score (9.5 vs. 7.7, *p* < 0.001) compared to parents of children undergoing elective surgery (*n* = 66). Parents in the emergent surgery group also reported higher anxiety scores on the HADS questionnaire (7.9 vs. 6.5, *p* = 0.009) and higher perceived stress and total score on the PSS-10 survey (7.8 vs. 5.6, *p* = 0.046) (10.5 vs. 9.1, *p* = 0.047), respectively. A significantly higher proportion of parents in the emergent surgery group were concerned about restricted visitation policies (*p* = 0.013) and reported delaying or considering delaying their child’s surgery due to the pandemic (*p* = 0.036). The results demonstrate heightened concerns, anxiety, and stress among parents of children undergoing emergency surgery during the COVID-19 pandemic. Healthcare providers should address parental concerns, provide clear communication, and ensure adequate support for families. Recommendations include enhancing information about COVID-19 precautions, reassuring parents about personal protective equipment availability, and facilitating family support within visitation restrictions.

## 1. Introduction

The coronavirus disease 2019 (COVID-19) pandemic, caused by the severe acute respiratory syndrome coronavirus 2 (SARS-CoV-2), has had an unprecedented impact on global health and the healthcare system. Although COVID-19 predominantly affects adults, children are not spared from the virus, and it has led to significant consequences on pediatric care, including surgery, as it is believed that COVID-19 has different effects on children and adults [1,2].

Large demographic research on patients validated these hypotheses by demonstrating that the percentage of cases in a severe state was much lower than in the general public. COVID-19 has a predisposition for atypical presentation in children, as shown by the large percentage of asymptomatic patients and the frequent lack of conventional symptoms [3]. In the same trial, only three children with preexisting diseases were brought to the intensive care unit, corroborating prior results of lower COVID-19 severity and better outcomes in the pediatric population [4].

The discrepancies between pediatric and adult patients may be attributable to the immaturity of the immune system and variations in the expression of the viral cell receptor in children [5,6]. Despite the absence of widespread testing for COVID-19, earlier studies indicated a substantially reduced presence of the illness in children compared to adults [7]; this could be linked to the lower overall exposure of children to infected individuals rather than to potential virus resistance [8]. Moreover, younger children have milder symptoms than teenagers [9]. However, this low incidence and absence of usual clinical signs raise concerns over the possible participation of children in the broad transmission of the virus.

Although children generally exhibit milder symptoms compared to adults, while some even remain asymptomatic [10], certain pediatric populations, such as those with underlying medical conditions, may be at a higher risk of developing severe complications from COVID-19 [11]. Furthermore, the emergence of the multisystem inflammatory syndrome in children (MIS-C), a rare but severe complication associated with SARS-CoV-2, has raised concerns among healthcare providers and parents alike [12].

The COVID-19 pandemic has led to a substantial reorganization of healthcare services, with elective surgeries postponed or canceled to prioritize urgent cases and allocate resources to the management of COVID-19 patients [13]. Additionally, the COVID-19 pandemic had a significant impact on the prevalence of some common pediatric disorders, such as otitis media with effusion, thus reducing the need for many scheduled surgeries, such as tympanostomy tube placement and adenoidectomy [14,15]. This has resulted in a backlog of pediatric surgical cases and increased anxiety among parents regarding the safety and timing of their child’s surgery. Additionally, parental concerns may be exacerbated by fear of nosocomial SARS-CoV-2 transmission during hospital visits or admissions, as well as the potential impact of delayed surgical interventions on their child’s health [16].

Understanding parental concerns in the pediatric surgery department during the COVID-19 pandemic is essential for healthcare providers to address these concerns effectively, ensure clear communication, and provide adequate support for both parents and children. Thus, the primary objective of the current study was to assess the extent and nature of parental concerns regarding pediatric surgical care during the COVID-19 pandemic, as well as to identify the factors associated with increased parental anxiety or concern in the context of pediatric surgery. Another objective was to provide recommendations for healthcare providers to address and alleviate parental concerns and improve the overall experience of pediatric surgical care during the ongoing pandemic and potential future outbreaks.

## 2. Materials and Methods

### 2.1. Study Design and Ethical Considerations

A cross-sectional study was designed at the Emergency Clinical Hospital for Children “Louis Turcanu”, a tertiary pediatric hospital in Timisoara, Romania. The study was conducted according to the guidelines of the Declaration of Helsinki and obtained the approval of the Ethical Commission of the involved institutions. The researchers involved in the current study gathered background and medical data from the hospital database and the associated patients’ paper records, where all treatments, procedures, and demographics were registered.

The inclusion criteria comprised the following particularities: (1) parents or legal guardians of pediatric patients requiring surgical care during the COVID-19 pandemic; (2) pediatric patients must have a confirmed surgical indication; (3) pediatric patients who have undergone or are scheduled to undergo elective or emergency surgery during the study period; and (4) participants being able to read, write, and understand the language in which the questionnaire is administered.

Parents or guardians were excluded for incomplete contact information or lack of consent to participate in the study. Other exclusion criteria comprised (1) parents or legal guardians who have previously participated in the study to avoid duplication of responses; (2) participants with cognitive or developmental disabilities that might affect their ability to understand the study or complete the questionnaires; (3) parents or pediatric patients who have been diagnosed with COVID-19 at the time of the survey, as this may introduce additional concerns and anxieties specific to their child’s COVID-19 infection rather than the surgery itself; and (4) patients experiencing severe emotional distress or mental health conditions that may interfere with their ability to provide accurate responses to the questionnaires or interfere with the anxiety and depression scores.

The sample size was determined using a convenience sampling method. The threshold for statistical significance was set at 0.05. A total of 300 parents or guardians were surveyed, of which 224 were accepted to participate. The surveys were completed online with the help of research assistants, while a total of 218 questionnaires were successfully completed, and 174 were included in the final analysis after excluding those with incomplete responses.

After the surveying period ended, the study cohort was stratified into two comparison groups. The first group of patients comprised parents of children who are scheduled for elective surgical procedures during the COVID-19 pandemic to help assess the parental concerns related to pediatric surgery when there is more time for preparation and planning and when the perceived risk of complications might be lower. The second study group comprised parents of pediatric patients who require emergency surgical procedures during the COVID-19 pandemic. Since emergency surgeries are those that need to be performed in case of imminent danger, the perceived risk of complications and anxiety might be higher.

### 2.2. Questionnaires and Variables

The current study assessed parental concerns regarding pediatric surgical care during the COVID-19 pandemic, factors associated with increased parental anxiety or concern, and recommendations for healthcare providers. The variables considered for analysis comprised the age and gender of patients and children, area of residence, marital status, level of income, level of education, employment status, COVID-19 vaccination status, number of children, type of pediatric surgical intervention, standardized questionnaire results, PCQ survey results, HADS survey results, and PSS-10 survey results. To assess parental concerns, we used the Parental Concerns Questionnaire (PCQ), the Hospital Anxiety and Depression Scale (HADS), and the Perceived Stress Scale (PSS-10).

The Parental Concerns Questionnaire (PCQ) is a tool designed to assess parental concerns related to pediatric surgery during the COVID-19 pandemic and includes both multiple-choice and open-ended questions, with reliability scores above 0.7 [17]. The PCQ consists of questions addressing the nature and extent of concerns, as well as demographic and clinical factors potentially associated with increased anxiety or concern. There are three domains analyzed by the PCQ questionnaire: (1) practical impact (items 1, 3, 5, 8, 10); (2) emotional impact (items 2, 4, 7, 9, 11); and (3) co-parent domain (items 6, 12, 13, 14, 15).

The Hospital Anxiety and Depression Scale (HADS) is a self-report scale designed to measure anxiety and depression in individuals in hospital or outpatient settings. It consists of fourteen items, with seven items assessing anxiety symptoms (HADS-A) and seven items assessing depression symptoms (HADS-D). Each item is scored on a 4-point scale, with higher scores indicating greater levels of anxiety or depression [18]. Cronbach’s alpha values for the HADS are considered to be acceptable to good, typically ranging from 0.70 to 0.90, depending on the studied population.

The Perceived Stress Scale (PSS-10) is a widely used self-report questionnaire designed to measure the degree to which individuals perceive situations in their lives as stressful [19]. The PSS-10 consists of 10 items, with each item rated on a 5-point Likert scale ranging from 0 (never) to 4 (very often). Participants are asked to indicate how often they have felt or thought a certain way in the past month. Higher scores indicate higher levels of perceived stress. Previous studies have reported Cronbach’s alpha values for the PSS-10 ranging from 0.74 to 0.89, indicating that the scale has reasonable internal consistency and reliability [20].

### 2.3. Statistical Analysis

Statistical analyses were performed using IBM SPSS Statistics for Windows, Version 26.0 (IBM Corp., Armonk, NY, USA). Descriptive statistics were used to summarize demographic and clinical characteristics, as well as parental concerns. The chi-square test or Fisher’s exact test, based on frequency assumptions, was used for the proportions of categorical variables. An independent-sample *t*-test or Mann–Whitney U test, as appropriate, was used for continuous variables based on the normality of data. Univariate logistic regression analyses were performed to identify factors associated with increased parental anxiety or concern. A *p*-value below 0.05 was considered statistically significant.

## 3. Results

### 3.1. Patients’ Background

At the end of the data collection and study period, a total of 66 parents were included in the group of children undergoing elective surgery, and 108 parents had children under emergency interventions. Table 1 presents the background characteristics of parents involved in the study, comparing those whose children underwent elective surgery (*n* = 66) to those whose children underwent emergent surgery (*n* = 108). The mean age of parents in the elective surgery group was 38.9 ± 6.7 years, while in the emergent surgery group, the mean age was 37.1 ± 5.9 years, although the difference was not statistically significant (*p* = 0.065).

Regarding gender, 92.4% (*n* = 61) of parents in the elective surgery group were female, compared to 87.0% (*n* = 94) in the emergent surgery group, with no significant difference between the groups (*p* = 0.268). The children’s mean age in the elective surgery group was 7.0 ± 2.4 years, significantly higher than the mean age of 5.1 ± 2.8 years in the emergent surgery group (*p* < 0.001). The proportion of female children in the elective surgery group was 42.4% (*n* = 28), while in the emergent surgery group, it was 46.3% (*n* = 50), with no significant difference between the two study groups (*p* = 0.618).

A higher percentage of parents in the elective surgery group lived in urban areas (66.7%, *n* = 44) compared to the emergent surgery group (49.1%, *n* = 53), and this difference was statistically significant (*p* = 0.023). There was no significant difference between the two study groups in terms of relationship status (married) (*p* = 0.594), level of income (average or higher) (*p* = 0.822), level of education (higher education) (*p* = 0.799), occupation (employed) (*p* = 0.559), or being a parent of an only child (*p* = 0.591). However, there was a significant difference in the proportion of parents who were vaccinated against COVID-19 between the elective surgery group (53.0%, *n* = 35) and the emergent surgery group (36.1%, *n* = 39) (*p* = 0.028).

Table 2 compares the types of pediatric surgical interventions during the COVID-19 pandemic between the elective surgery (*n* = 66) and emergent surgery (*n* = 108) groups. The distribution of surgical interventions across the two groups was not statistically significant (*p* = 0.301). In the elective surgery group, the types of interventions were as follows: digestive (47.0%, *n* = 31), neonate (3.0%, *n* = 2), urology (16.7%, *n* = 11), orthopedics (7.6%, *n* = 5), abdominal (21.2%, *n* = 14), and others (4.5%, *n* = 3). In the emergent surgery group, the distribution of interventions included digestive pathology (50.9%, *n* = 55), neonatal interventions (5.6%, *n* = 6), urological interventions (7.4%, *n* = 8), orthopedics (14.8%, *n* = 16), abdominal surgery (17.6%, *n* = 19), and other types of pathologies requiring surgical intervention (3.7%, *n* = 4). Although the proportions of each type of intervention varied between the elective and emergent surgery groups, the overall distribution was not statistically different between the two groups (*p*-value = 0.301).

### 3.2. Analysis of Unstandardized Questionnaires

A significantly higher proportion of parents in the elective surgery group (56.1%, *n* = 37) expressed concern about their child being exposed to COVID-19 during their surgical procedure or hospital stay compared to the emergent surgery group (38.0%, *n* = 41) (*p* = 0.019). Additionally, a significantly larger percentage of parents in the emergent surgery group (60.2%, *n* = 65) reported delaying or considering delaying their child’s surgery due to the ongoing COVID-19 pandemic, compared to the elective surgery group (43.9%, *n* = 29) (*p* = 0.036).

There was no significant difference between the groups in terms of feeling adequately informed about the precautions taken by the hospital to prevent COVID-19 transmission during their child’s surgery, with 39.4% (*n* = 26) in the elective surgery group and 37.0% (*n* = 40) in the emergent surgery group (*p* = 0.755). Similarly, there was no significant difference in concerns about the availability of personal protective equipment (PPE) for healthcare providers during their child’s surgery (*p* = 0.329) or concerns about the potential impact of COVID-19 on the quality of care their child would receive during their surgical procedure (*p* = 0.758).

However, a significantly higher proportion of parents in the emergent surgery group (76.9%, *n* = 83) were anxious about the possibility of restricted visitation policies affecting their ability to support their child during their hospital stay, compared to the elective surgery group (59.1%, *n* = 39) (*p* = 0.013). There was no significant difference between the groups regarding whether the COVID-19 pandemic had affected their decision-making process concerning their child’s surgical care (*p* = 0.509), as seen in Table 3.

### 3.3. Analysis of Standardized Questionnaires

Parents in the emergent surgery group reported a significantly higher level of practical impact (median = 3.4, interquartile range (IQR) = 1.4–3.9) compared to the elective surgery group (median = 2.8, IQR = 1.6–3.3) (*p* < 0.001). Similarly, the emotional impact scores were significantly higher in the emergent surgery group (median = 2.7, IQR = 1.6–3.6) compared to the elective surgery group (median = 2.2, IQR = 1.1–3.0) (*p* = 0.002). There was no significant difference between the groups in terms of the co-parent domain, with a median score of 2.0 (IQR = 1.3–2.9) in the elective surgery group and 2.3 (IQR = 1.4–3.0) in the emergent surgery group (*p* = 0.266).

**Figure 1:**
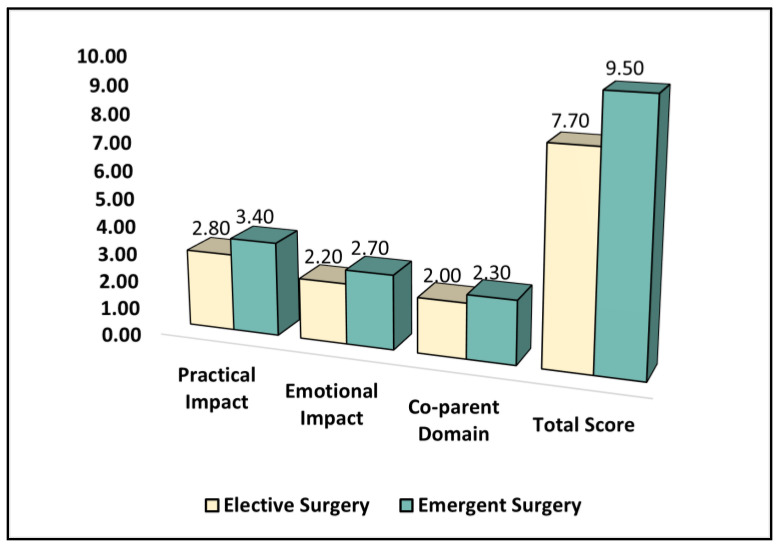
A comparison of the PCQ survey results.

However, the total PCQ score, which represents the overall level of concern or anxiety among parents, was significantly higher in the emergent surgery group (median = 9.5, IQR = 5.1–12.9) compared to the elective surgery group (median = 7.7, IQR = 4.5–9.2) (*p* < 0.001), as seen in Table 4 and Figure 1. This indicates a higher level of concern and anxiety among parents whose children underwent emergent surgery during the COVID-19 pandemic compared to those whose children underwent elective surgery.

Parents in the emergent surgery group reported a significantly higher mean score for anxiety (mean = 7.9, SD = 3.5) compared to the elective surgery group (mean = 6.5, SD = 3.2) (*p* = 0.009). However, there was no significant difference between the groups in terms of the mean score for depression, with a mean score of 6.1 (SD = 3.5) in the elective surgery group and 6.9 (SD = 3.1) in the emergent surgery group (*p* = 0.118), as described in Table 5.

**Figure 2:**
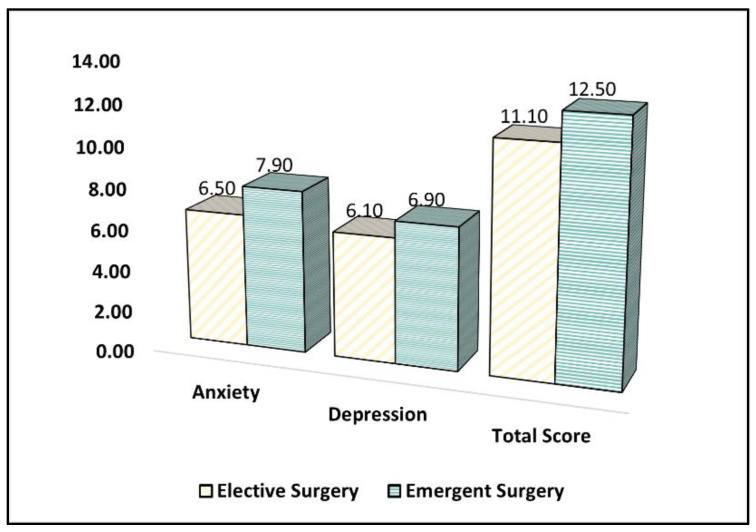
A comparison of the HADS survey results.

The total HADS score, which represents the overall level of anxiety and depression, showed no significant difference between the groups, with a mean score of 11.1 (SD = 5.8) in the elective surgery group and 12.5 (SD = 5.2) in the emergent surgery group (*p* = 0.101), as presented in Figure 2. Although the total score did not show a significant difference, the increased anxiety scores indicate that parents whose children underwent emergent surgery during the COVID-19 pandemic experienced higher levels of anxiety compared to those whose children underwent elective surgery.

Table 6 presents the comparison of the Perceived Stress Scale (PSS-10) survey results between parents whose children underwent elective surgery and those whose children underwent emergent surgery during the COVID-19 pandemic. Parents in the emergent surgery group reported a significantly higher mean score for positive items (mean = 7.8, standard deviation (SD) = 3.9) compared to the elective surgery group (mean = 5.6, SD = 3.7) (*p* = 0.046). However, there was no significant difference between the groups in terms of the mean score for negative items, with a mean score of 5.9 (SD = 2.8) in the elective surgery group and 6.6 (SD = 3.1) in the emergent surgery group (*p* = 0.135).

**Figure 3:**
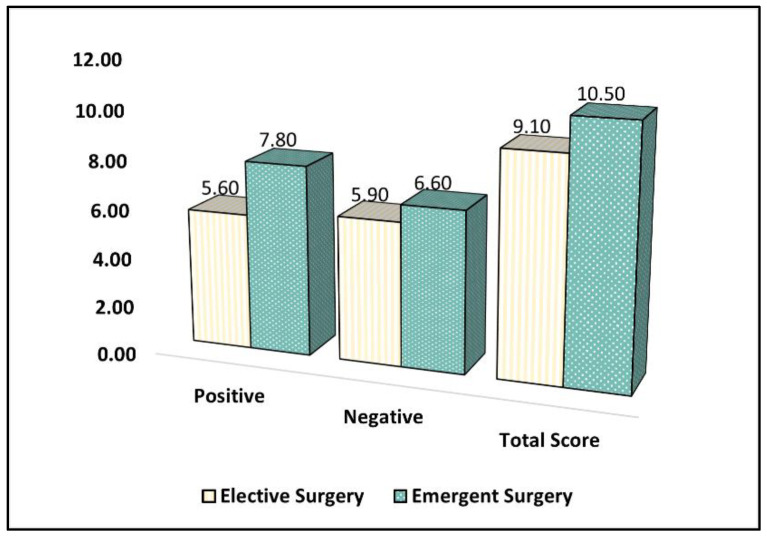
A comparison of the PSS-10 survey results.

The total PSS-10 score, which represents the overall level of perceived stress, was significantly higher in the emergent surgery group (mean = 10.5, SD = 4.6) compared to the elective surgery group (mean = 9.1, SD = 4.3) (*p* = 0.047), as seen in Figure 3. This indicates that parents whose children underwent emergent surgery during the COVID-19 pandemic experienced higher levels of perceived stress compared to those whose children underwent elective surgery.

### 3.4. Correlation and Risk Factor Analysis

A Pearson correlation analysis was performed between the three standardized questionnaires in the elective surgery group and the emergent surgery group, respectively. All three questionnaires show significant positive correlations in both the elective and emergent surgery groups, suggesting that higher levels of parental concerns (PCQ) are associated with higher levels of anxiety and depression (HADS) and perceived stress (PSS-10), as described in Table 7. The strongest correlation in both groups is between the HADS and PSS-10, indicating that anxiety and depression are closely related to perceived stress in both elective and emergent surgery contexts.

Table 8 presents the factors associated with increased parental anxiety or concern in the pediatric surgery department during the COVID-19 pandemic. The age of the children (per 1-year increase) was significantly associated with decreased parental anxiety or concern (β = −0.36, 95% CI = −0.60 (−0.94), *p* = 0.002). Parents with a higher level of education compared to those with lower education reported significantly increased anxiety or concern (β = 1.62, 95% CI = 1.06–4.15, *p* = 0.013). Being a parent of an only child was also significantly associated with increased anxiety or concern (β = 1.45, 95% CI = 1.12–4.75, *p* = 0.007).

The Parental Concerns Questionnaire (PCQ) total score and the Perceived Stress Scale (PSS-10) total score were significantly associated with increased parental anxiety or concern, with values of β = 2.92 (95% CI = 1.33–5.68, *p* < 0.001) and β = 2.56 (95% CI = 1.12–6.06, *p* = 0.001), respectively. The Hospital Anxiety and Depression Scale (HADS) total score, however, was not significantly associated with increased parental anxiety or concern (β = 1.30, 95% CI = 0.97–2.91, *p* = 0.205). The remaining variables, including the age of parents, parents’ gender, children’s gender, area of residence, relationship status, level of income, occupation, and COVID-19 vaccination status, were not significantly associated with increased parental anxiety or concern.

In Table 9, the results indicate the association between different types of interventions and increased parental anxiety or concern during the COVID-19 pandemic. Digestive interventions were significantly associated with heightened parental anxiety or concern (β = 1.66, 95% CI: 1.05–4.09, *p* = 0.010), as were neonate interventions (β = 1.94, 95% CI: 1.12–3.18, *p* = 0.008). However, the associations for urology (β = 1.25, 95% CI: 0.62–2.51, *p* = 0.324), orthopedics (β = 1.02, 95% CI: 0.39–1.66, *p* = 0.491), and abdominal (β = 1.47, 95% CI: 0.92–2.50, *p* = 0.108) interventions were not statistically significant. Thus, the findings suggest that during the COVID-19 pandemic, parents of children undergoing digestive and neonate surgeries experienced significantly higher levels of anxiety or concern compared to other types of pediatric surgeries.

## 4. Discussion

### 4.1. Literature Analysis

The current study aimed to assess the nature and extent of parental concerns regarding pediatric surgical care during the COVID-19 pandemic and identify the factors associated with increased parental anxiety or concern. Our findings indicate that parents whose children underwent emergent surgery during the pandemic experienced higher levels of concern, anxiety, and perceived stress compared to those whose children underwent elective surgery. These results are consistent with previous studies reporting increased anxiety among parents of children undergoing surgery during the COVID-19 pandemic.

In the study by Former et al. conducted during the first wave of the COVID-19 pandemic, it was found that parents with children awaiting elective, non-emergent surgery experienced significant psychosocial distress [19]. The distress was not necessarily related to SARS-CoV-2 transmission but rather to prolonged wait times and hospital restrictions. In a previous study by Miller et al., the authors found that 95% of parents felt waiting for their child’s surgery caused emotional distress, with half of them perceiving their child’s health as deteriorating [21]. Another study investigated the impact of the COVID-19 pandemic on parental anxiety levels and explored the need for new forms of parenting. Using a quantitative descriptive method and the GAD-7 instrument, the results showed that 63.08% of parents experienced moderate to severe anxiety. Furthermore, parental satisfaction with current parenting forms was low at 67.12%, while interest in new parenting forms was very high at 98.51% [22].

Our study found that the age of the children was significantly lower in the emergent surgery group compared to the elective surgery group (5.1 ± 2.8 vs. 7.0 ± 2.4, *p* < 0.001). Other studies have also reported the association between children’s age and parental anxiety, stress, and concerns. For example, a study by Barkmann et al. [23] found that younger children’s parents had higher levels of anxiety and concerns before their child’s surgery. Although our study did not find a significant difference in parental anxiety or concerns based on gender, other studies have reported that mothers tend to have higher anxiety and stress levels compared to fathers in the context of their children’s surgeries [24].

Two factors contributing to increased psychosocial distress for parents were hospital restrictions and the quality of communication with the healthcare team. Parents experienced distress due to the inability to have a second support person with them during the perioperative period and poor communication with the healthcare team [25]. The greatest burden of distress was experienced by parents of children who were required to travel from out of province and thus needed to self-isolate, had multiple delays, and had a perceived more severe disease with a higher risk of developmental delay [26]. Additionally, parents who were more familiar with their child’s diagnosis and pending procedure expressed lower levels of distress. Additionally, studies suggest that improved parent education during initial pre-operative consultations may be an avenue for targeted support in reducing distress [20].

A significant proportion of parents expressed concern about their child being exposed to COVID-19 during their surgical procedure or hospital stay, particularly in the elective surgery group. This finding is consistent with existing literature that reports heightened concern among parents about the risk of COVID-19 infection in healthcare settings [27]. The difference in concern between elective and emergent surgery groups may be due to the perceived level of control over the situation; parents of children undergoing elective surgery may feel they have more choice in the timing of the procedure and thus may have heightened concerns about the risks of proceeding during the pandemic.

Our study also found that a significantly larger percentage of parents in the emergent surgery group reported delaying or considering delaying their child’s surgery due to the ongoing COVID-19 pandemic. This is in line with previous research that suggests parents may weigh the risks and benefits of proceeding with surgery during a pandemic and may opt to delay procedures due to concerns about infection risk [20,28]. However, our study found a significant difference in COVID-19 vaccination status between the elective and emergent surgery groups (*p* = 0.028). Parents in the elective surgery group had a higher percentage of COVID-19 vaccination. Although we did not find a direct association between vaccination status and the questionnaire scores, other studies have reported that vaccination status can influence parental anxiety and stress levels during the COVID-19 pandemic [29].

Interestingly, there was no significant difference between the groups in terms of feeling adequately informed about the precautions taken by the hospital to prevent COVID-19 transmission during their child’s surgery. This suggests that healthcare providers may have been effective in communicating the measures in place to reduce the risk of infection during surgical procedures [30,31]. However, a significantly higher proportion of parents in the emergent surgery group were anxious about the possibility of restricted visitation policies affecting their ability to support their children during their hospital stay. This finding highlights the importance of family-centered care and the role of healthcare providers in addressing parental concerns and ensuring adequate support for families during hospitalization [32].

The increased anxiety and stress levels among parents in the emergent surgery group may be attributed to several factors, including the urgent nature of the procedure, concerns about potential complications, and uncertainty about the outcome. Previous studies have reported that parental anxiety and stress are associated with their child’s surgery type, with higher levels observed among parents of children undergoing emergent surgery compared to elective surgery [33]. Our study also found that parents in the emergent surgery group had significantly higher PCQ practical impact and emotional impact scores, indicating greater concerns related to the logistics of their child’s surgery and the emotional toll of the experience. These findings emphasize the need for healthcare providers to address the practical and emotional needs of families in the context of pediatric surgery during the pandemic.

Healthcare providers can address and alleviate parental concerns and improve the overall experience of pediatric surgical care during the ongoing pandemic and potential future outbreaks by enhancing communication and providing comprehensive information on COVID-19 precautions and personal protective equipment availability. They should also work with hospital administration to facilitate family support within visitation restrictions, offer access to mental health professionals, and utilize telehealth services for consultations and follow-up care [34]. Furthermore, healthcare providers should educate parents on the importance of timely surgical intervention, encourage feedback and collaboration, and prepare for future outbreaks by developing and implementing protocols and guidelines for pediatric surgery departments that prioritize patient and staff safety while ensuring continuity of care.

### 4.2. Strengths and Limitations

The current study has several strengths. First, it adopted a cross-sectional design that allowed the researchers to capture a snapshot of the population during the COVID-19 pandemic, providing valuable insights into the concerns and anxiety levels of parents whose children required pediatric surgical care during this period. This design helped uncover specific factors related to heightened anxiety and concern among parents, facilitating the development of recommendations for healthcare providers to address these concerns. Second, the use of standardized questionnaires, such as the PCQ, HADS, and PSS-10, allowed for more systematic and consistent measurement of parental concerns and anxiety levels and enabled comprehensive and reliable data collection, which ultimately enhanced the study’s overall validity.

However, the study also has some limitations, such as the use of convenience sampling, which may have introduced sampling bias and limited the generalizability of the findings to the broader population of parents with children requiring surgical care. Additionally, the sample size, while adequate for the cross-sectional design, may not be large enough to detect more subtle differences between the elective and emergent surgery groups, potentially affecting the study’s statistical power. The study also relied on self-reported data from the parents, which could introduce response bias or inaccuracies due to recall bias. Lastly, the exclusion of parents or pediatric patients diagnosed with COVID-19 at the time of the survey may have limited the understanding of how COVID-19 itself influenced parental concerns and anxiety levels during the pandemic.

## 5. Conclusions

The current study highlights the increased concerns, anxiety, and perceived stress among parents of children undergoing pediatric surgery during the COVID-19 pandemic, particularly in the emergent surgery group. The age of the children, parental education level, and being a parent of an only child were significant predictors of increased anxiety or concern. Additionally, the total scores of the Parental Concerns Questionnaire (PCQ) and Perceived Stress Scale (PSS-10) were significantly associated with increased parental anxiety or concern, while the Hospital Anxiety and Depression Scale (HADS) total score was not significantly associated with it.

In terms of surgical interventions, digestive and neonatal interventions were significantly associated with increased parental anxiety, while urology, orthopedics, and abdominal interventions showed no significant association. Healthcare providers should be aware of these concerns and work to address them through clear communication, family-centered care, and the provision of adequate support for both parents and children. Further research is needed to explore the long-term effects of the pandemic on parental concerns in pediatric surgery and develop effective strategies to alleviate these concerns and improve the overall experience of pediatric surgical care during future outbreaks.

## Figures and Tables

**Table 1:** A comparison of the background characteristics of parents involved in the study.

Variables	Elective (*n* = 66)	Emergent (*n* = 108)	*p*-Value
Age of parents (mean ± SD)	38.9 ± 6.7	37.1 ± 5.9	0.065
Parents’ gender (female)	61 (92.4%)	94 (87.0%)	0.268
Age of children (mean ± SD)	7.0 ± 2.4	5.1 ± 2.8	<0.001
Children’s gender (female)	28 (42.4%)	50 (46.3%)	0.618
Area of residence (urban)	44 (66.7%)	53 (49.1%)	0.023
Relationship status (married)	63 (95.5%)	101 (93.5%)	0.594
Level of income (average or higher)	20 (30.3%)	31 (28.7%)	0.822
Level of education (higher education)	22 (33.3%)	34 (31.5%)	0.799
Occupation (employed)	38 (57.6%)	67 (62.0%)	0.559
COVID-19 vaccinated (yes)	35 (53.0%)	39 (36.1%)	0.028
Only child parent (yes)	37 (56.1%)	65 (60.2%)	0.591

Data reported as *n* (frequency) and calculated using the chi-square test and Fisher’s exact test, unless specified differently.

**Table 2:** A comparison between types of pediatric surgical interventions during the COVID-19 pandemic.

Type of Intervention	Elective (*n* = 66)	Emergent (*n* = 108)	*p*-Value
			0.301
Digestive	31 (47.0%)	55 (50.9%)	
Neonate	2 (3.0%)	6 (5.6%)	
Urology	11 (16.7%)	8 (7.4%)	
Orthopedics	5 (7.6%)	16 (14.8%)	
Abdominal	14 (21.2%)	19 (17.6%)	
Others	3 (4.5%)	4 (3.7%)	

Data reported as *n* (frequency) and calculated using the chi-square test and Fisher’s exact test, unless specified differently.

**Table 3:** Unstandardized questionnaire results.

Questions (Yes/No)	Elective (*n* = 66)	Emergent (*n* = 108)	*p*-Value
Are you concerned about your child being exposed to COVID-19 during their surgical procedure or hospital stay?	37 (56.1%)	41 (38.0%)	0.019
Have you delayed or considered delaying your child’s surgery due to the ongoing COVID-19 pandemic?	29 (43.9%)	65 (60.2%)	0.036
Do you feel adequately informed about the precautions taken by the hospital to prevent COVID-19 transmission during your child’s surgery?	26 (39.4%)	40 (37.0%)	0.755
Are you worried about the availability of personal protective equipment (PPE) for healthcare providers during your child’s surgery?	19 (28.8%)	24 (22.2%)	0.329
Do you have concerns about the potential impact of COVID-19 on the quality of care your child will receive during their surgical procedure?	11 (16.7%)	20 (18.5%)	0.758
Are you anxious about the possibility of restricted visitation policies affecting your ability to support your child during their hospital stay?	39 (59.1%)	83 (76.9%)	0.013
Do you feel that the COVID-19 pandemic has affected your decision-making process regarding your child’s surgical care?	8 (12.1%)	17 (15.7%)	0.509

Data reported as *n* (frequency) and calculated using the chi-square test and Fisher’s exact test, unless specified differently.

**Table 4:** Analysis of the PCQ survey results.

Items (Median, IQR)	Elective (*n* = 66)	Emergent (*n* = 108)	*p*-Value
Practical impact	2.8 (1.6–3.3)	3.4 (1.4–3.9)	<0.001
Emotional impact	2.2 (1.1–3.0)	2.7 (1.6–3.6)	0.002
Co-parent domain	2.0 (1.3–2.9)	2.3 (1.4–3.0)	0.266
Total score	7.7 (4.5–9.2)	9.5 (5.1–12.9)	<0.001

Data reported as *n* (frequency) and calculated using the chi-square test and Fisher’s exact test, unless specified differently; IQR—interquartile range; PCQ—Parental Concerns Questionnaire (higher scores represent a higher level of concern or anxiety among parents).

**Table 5:** A comparison of the HADS survey results.

Items (Mean ± SD)	Elective (*n* = 66)	Emergent (*n* = 108)	*p*-Value
Anxiety	6.5 ± 3.2	7.9 ± 3.5	0.009
Depression	6.1 ± 3.5	6.9 ± 3.1	0.118
Total score	11.1 ± 5.8	12.5 ± 5.2	0.101

Data reported as mean ± SD and calculated using Student’s *t*-test; HADS—Hospital Anxiety and Depression Scale (higher scores indicate greater levels of anxiety or depression).

**Table 6:** A comparison of the PSS-10 survey results.

Items (Mean ± SD)	Elective (*n* = 66)	Emergent (*n* = 108)	*p*-Value
Positive	5.6 ± 3.7	7.8 ± 3.9	0.046
Negative	5.9 ± 2.8	6.6 ± 3.1	0.135
Total score	9.1 ± 4.3	10.5 ± 4.6	0.047

Data reported as mean ± SD and calculated using Student’s *t*-test; PSS-10—Perceived Stress Scale (higher scores indicate higher levels of perceived stress).

**Table 7:** Pearson correlation coefficients between the PCQ, HADS, and PSS-10 scores for the elective and emergent surgery groups.

Correlations (Total Score)	Elective (*n* = 66)	Emergent (*n* = 108)
PCQ and HADS	r = 0.652, *p* < 0.001	r = 0.585, *p* < 0.001
PCQ and PSS-10	r = 0.720, *p* < 0.001	r = 0.633, *p* < 0.001
HADS and PSS-10	r = 0.794, *p* < 0.001	r = 0.546, *p* < 0.001

HADS—Hospital Anxiety and Depression Scale; PSS-10—Perceived Stress Scale; PCQ—Parental Concerns Questionnaire.

**Table 8:** Factors associated with increased parental anxiety or concern.

Variables	β	95% CI	*p*-Value
Age of parents (per 1-year increase)	−0.88	−0.32–0.96	0.328
Age of children (per 1-year increase)	−0.36	−0.60–(−0.94)	0.002
Parents’ gender (female vs. male)	1.85	0.52–5.53	0.351
Children’s gender (female vs. male)	0.92	0.08–2.10	0.219
Area of residence (urban vs. rural)	1.04	0.79–1.66	0.594
Relationship status (married vs. unmarried)	−0.95	0.38–0.91	0.090
Level of income (average or above vs. below average)	1.24	0.92–2.28	0.136
Level of education (higher education vs. lower education)	1.62	1.06–4.15	0.013
Occupation (employed vs. unemployed)	−0.86	−1.27–1.09	0.447
COVID-19 vaccinated (yes vs. no)	0.61	0.27–1.94	0.142
Only child parent (yes vs. no)	1.45	1.12–4.75	0.007
PCQ total score	2.92	1.33–5.68	<0.001
HADS total score	1.30	0.97–2.91	0.205
PSS-10 total score	2.56	1.12–6.06	0.001

β—standardized beta coefficient; CI—confidence interval.

**Table 9:** Interventions associated with increased parental anxiety or concern.

Type of Intervention	β	95% CI	*p*-Value
Digestive	1.66	1.05–4.09	0.010
Neonate	1.94	1.12–3.18	0.008
Urology	1.25	0.62–2.51	0.324
Orthopedics	1.02	0.39–1.66	0.491
Abdominal	1.47	0.92–2.50	0.108

β—standardized beta coefficient; CI—confidence interval.

## Data Availability

The data presented in this study are available upon request from the corresponding author.
